# Polypharmacy in Zoological Medicine

**DOI:** 10.3390/pharmaceutics9010010

**Published:** 2017-02-22

**Authors:** Robert P. Hunter, Ramiro Isaza

**Affiliations:** 1 One Medicine Consulting, Olathe, KS 66062, USA; 2 College of Veterinary Medicine, University of Florida, Gainesville, FL 23610, USA; isazar@ufl.edu

**Keywords:** polypharmacy, zoological medicine, pharmacology, pharmacokinetics, minor species

## Abstract

Polypharmacy is a term that describes the inappropriate, concurrent use of multiple drugs in an individual patient. Zoological medicine practitioners must take approved agents (veterinary or human) and extrapolate their use to non-approved species often with little species-specific pharmacological evidence to support their decisions. When considering polypharmacy, even less information exists concerning multi-drug pharmacokinetics, pharmacodynamics, or potential drug-drug interactions in non-domestic species. Unfortunately, captive, zoological species are susceptible, just like their domestic counterparts, to chronic diseases and co-morbidities that may lead to the usage of multiple drugs. Polypharmacy is a recognized and important issue in human medicine, as well as an emerging issue for veterinarians; thus, this paper will discuss the novel, potential risks of polypharmacy in zoological medicine. Hopefully, this discussion will help bring the attention of veterinarians to this issue and serve as an interesting discussion topic for pharmacologists in general.

## 1. Introduction

Polypharmacy describes the inappropriate, concurrent use of multiple drugs in an individual patient. This differs from the common human therapy definition of polypharmacy which is the concurrent administration of ≥6 drugs to a single patient. However, even in human medicine, the arbitrary assignment of a “>n” value may not be appropriate [1]. For the purposes of this manuscript, a working definition is the use of more medications than is medically necessary or optimal for the overall treatment or welfare of the animal patient. Generally, the more drugs a patient takes, the greater the risk of adverse events and drug-drug interactions.

The negative effects of polypharmacy are a consideration in any species and should be a concern to all prescribers of medications. Underlying many cases of polypharmacy is a temptation toward using several of the available drugs to address a given clinical problem. This may be an extension of the fallacy that if a small dose works, a larger dose is better, or if one drug works, an additional drug should be better. The often-unrecognized consequences of the use of multiple drugs are the cumulative adverse effects that occur in the patient. When mixing drugs, the prescriber and pharmacist need to have a clear understanding of both the expected effects of each drug used and the potential novel adverse events resulting from combining drugs.

Zoological veterinary medicine is the practice of all aspects of veterinary medicine on non-domestic species in both wild and captive situations. Here, non-domestic species include all animals except dogs, cats, horses, cows, sheep, goats, swine, and poultry [2], and ranges from invertebrates to mammals. Unfortunately, veterinarians practicing zoological medicine have very few species-specific, labeled drugs and must take approved agents from the companion animal or human pharmacopeia and extrapolate their use to non-approved species [3]. While there may be information on the label for potential drug-drug interactions, efficacy, and pharmacokinetics for the approved labeled species, generally no label information exists for the non-domestic species requiring treatment. This review will attempt to provide novel insight into polypharmacy in zoological medicine and highlight what some of the potential risks are when it is employed in this wide variety of species.

## 2. Why Is Polypharmacy of Particular Relevance to Zoological Medicine?

Veterinary practitioners of zoological medicine in the United States have most of the approved drugs for both human and domestic animals available. The indications, pharmacokinetics, pharmacodynamics, and toxicology of pharmaceutics are all well-established through strict regulatory procedures for the labeled species. This information is available in the product labels, peer reviewed literature, and formularies. Unfortunately, all of this combined information is usually species specific and is generally not available for non-domestic species that need treatment. The many different species-related physiological factors, such as body temperature regulation, blood flow, intrinsic hepatic metabolism, and renal physiology, lead to variations in the clearance of pharmaceuticals between and within species. Differences in gastrointestinal physiology can and do lead to potential variation in drug absorption, metabolism, and excretion. When one considers the anatomical differences between monogastrics (dog, cat, or swine), hindgut fermenters (rodents, rabbits, horses, or elephants), foregut fermenters (Colobus monkeys, camelids, and kangaroos), and ruminants (cattle, goats, sheep, or antelope), the potential differences are staggering and support the concept that bioavailability is not comparable across even mammalian species [4,5]. These species differences can affect the pharmacokinetics, pharmacodynamics, and toxic properties of any particular drug [6]. This was illustrated in a study of the pharmacokinetics of ketoprofen in elephants compared to those reported in horses, which identified multiple clinically relevant differences [7].

By extension, this limitation in our species-specific understanding of basic drug metabolism and pharmacokinetic parameters in non-domestic species hinders not only individual drug usage, but also heightens the deleterious impact of polypharmacy in zoological medicine. With more drugs commercially available, zoological medicine veterinarians may become more likely to use multiple drugs for the treatment of complex clinical conditions. Unfortunately, this usage of multiple drugs can be associated with unexpected adverse effects, a situation that advocates for both the discussion and consideration of polypharmacy in zoological medicine.

## 3. When Does Polypharmacy Occur in Zoological Medicine? 

As in veterinary medicine of domestic species, zoological medicine polypharmacy is common in patients with chronic diseases, geriatric animals with multiple problems, and in balanced anesthetic protocols using multiple drugs. Here, in several examples, we use elephants to illustrate the problem of polypharmacy inherent in the practice of zoological medicine [8].

### 3.1. Chronic Disease

In human medicine, polypharmacy is a recognized and common problem in the treatment of chronic diseases. For example, among United States adults with arthritis, 28% were found to be taking concurrent prescription drugs from six or more drug classes. This polypharmacy was negatively associated with physical performance scores among adults with arthritis [9]. Polypharmacy is considered an important and increasing challenge in human clinical practice because of its association with adverse outcomes [1].

The benefits and risks of polypharmacy in veterinary practice have been addressed only in a very limited fashion starting in the late 1970s [10,11]. Although in human medicine drug-drug interactions are an integral part of the United States (and numerous other countries’) drug approval process, this is not the case for veterinary medicine, where these interactions are typically only addressed post-approval when reported via pharmacovigilance. Reporting of adverse effects in non-domestic species, however, has been negligible.

Furthermore, pharmacokinetic studies or pharmaceutical plasma concentration data for the treatment of chronic conditions in non-domestic animals are very limited. Most of the available studies have focused on analgesic or anti-inflammatory agents, and none have evaluated the long term adverse effects of medications used for treating chronic conditions [8,12]. Because of the relative dearth of species-specific pharmacokinetic and toxicological data for long-term therapy, zoological medicine dosage regimens are often based on the weak assumptions that pharmaceutical compounds evaluated for single dose pharmacokinetics will be safe when used chronically, and that drugs safely administered chronically to domestic species will be similarly safe when used concurrently with other drugs in non-domestic species.

Nevertheless, zoological medicine practitioners often need to treat chronic conditions for extended periods of time. Arthritis is such a condition, requiring long term pain management to decrease disease severity and improve patient welfare. Arthritic elephants, for example, are often treated concurrently with a variety of nonsteroidal anti-inflammatory drugs and opioid analgesics. However, because of the recognized adverse effects of these agents in domestic species, appropriate therapeutic regimens are critical to appropriate treatment [13]. In another case, an adult African elephant being treated for subcutaneous edema was given a combination of flunixin meglumine and six other drugs that ended with post mortem evidence of renal papillary necrosis and gastrointestinal mucosal damage [14].

An example of how the human treatment information can be interpreted and potentially misused is the example of *Mycobacterium tuberculosis* treatment in elephants. Unlike cattle and other livestock in the United States, elephants are recognized for their rarity and value and are treated rather than culled. Guidelines for drug administration in pachyderms have been derived from those established for humans without prior testing in elephants [15]. As in humans, these protocols mandate the usage of multiple concurrent first line anti-tuberculosis drugs for an extended period. As a result, numerous adverse effects have occurred in the majority of elephants treated [16]. In many elephants, these were severe enough that treatment needed to be temporarily discontinued. Reported adverse effects include anorexia, depression, diarrhea, kidney and liver insults, blepharospasm, and death. Wiedner and Schmitt [16] reported that the high incidence of severe adverse effects was most likely related to the doses of drugs required to achieve circulating concentrations comparable to those in humans. Another possible explanation of the serious adverse effects is polypharmacy and unrecognized, elephant specific, drug-drug interactions of these antimicrobial agents.

These examples highlight that although several of the drugs used in these elephants have been evaluated with single dose pharmacokinetic studies, none was ever evaluated either for chronic or concurrent usage [7,8,13]. The determination of effectiveness of these multi-modal drug regimens is generally based on clinical response to treatment without the evaluation for subtle negative side effects. As a result, the potential for significant renal, gastric, and hepatic injury may often be underestimated and overlooked, highlighting the need to evaluate, and then publish the chronic effects of polypharmacy in non-domestic species.

### 3.2. Geriatric Animals

Geriatric patients of any species tend to have more medical problems and thus need more drugs in their treatment. A significant amount of data examining polypharmacy in the geriatric human population have identified several concerning issues [1,17,18]. Several studies have documented inappropriate prescription rates ranging from 22.5% and 28.4% in elderly human patients [1,17,18]. In one study, 23.9% of elderly patients were found to be receiving at least one potentially inappropriate prescription [17].

As in human medicine, effective geriatric care in veterinary medicine often necessitates multiple drugs to treat age-related co-morbidities. In the geriatric non-domestic animal patient, age related differences in drug metabolism add further confounding effects. Here too, the risks of polypharmacy require consideration although the subject has only been minimally addressed in the veterinary literature.

In well-managed populations of captive animals, individual animals tend to live longer. Thus, the zoological medicine veterinarian is often responsible for the treatment and welfare of numerous geriatric non-domestic animals. This sub-set of geriatric animals often has multiple co-morbidities, in addition to age-related changes in metabolism that may be difficult to identify.

For example, this is especially important in long lived elephants where the demographics of the captive population have become predominantly geriatric animals [19]. Older elephants commonly have concurrent chronic arthritis, dental malocclusions, reproductive disorders of senescence, and age related subclinical renal or hepatic function declines. In zoological medicine, many older elephants are receiving multiple drug treatments for the management of these geriatric conditions. A given elephant may be prescribed chronic ketoprofen for mild arthritis, intermittent ceftiofur for pododermitis, and butorphanol for occasional colic episodes. Although all these drugs have been evaluated for single dose pharmacokinetics, no studies have evaluated the net effects of these types of drug combinations [7,13,20].

Thus, periodic and careful pharmacological case review is needed to manage non-domestic geriatric patients requiring multiple drugs, with particular attention paid to new clinical problems that may be inadvertently caused by drug-drug interactions. Also needed are publications documenting the post-mortem evaluations of geriatric non-domestic species with consideration of the multiple drugs they received near the ends of their lives.

### 3.3. Anesthesia of Non-Domestic Species

Balanced anesthesia uses multiple drugs to produce the combined effects of unconsciousness, muscle relaxation, and analgesia, in conjunction with hemodynamic stability and, ideally, reversibility. Such cocktail combinations may include three or more different drugs administered sequentially or concurrently throughout the anesthetic event. The goal of balanced anesthesia is not to use lower doses of each drug but to titrate the drugs together to produce the desired anesthetic effects. Balanced anesthesia is therefore by definition a form of polypharmacy. However, with each drug the probability of adverse physiological effects increases. When these drug cocktails are then combined with other non-anesthetic drugs administered for chronic or geriatric conditions, the full range of polypharmacy concerns can further complicate the anesthetic procedure. Furthermore, in these multidrug protocols, determining which drug or set of drugs precipitated the adverse anesthetic event becomes extremely difficult.

Historically, single drug protocols using dissociative anesthetic drugs (i.e., ketamine) or inhalant anesthesia (i.e., isoflurane) were the mainstay of veterinary anesthetic protocols. Although effective, these protocols were often associated with qualitatively unsatisfactory anesthetic events as well as unacceptable side-effects due to relative over dosages of the single drug. As in human medicine, this type of anesthesia evolved over time with the recognition that no single ideal anesthetic agent exists, and that improved anesthetic protocols can be developed using combinations of different drugs. Although formularies and textbooks are available to help the veterinarian select species-specific anesthetic protocols, ultimately the choice of drugs in a balanced anesthesia protocol for a non-domestic species becomes a combination of art and science, modified with the goal of having a safe and effective anesthetic event.

Because most non-domestic species are uncooperative regarding restraint and manipulation, zoological medicine veterinarians often have to anesthetize or sedate a large proportion of their patients for medical procedures. However, with minimal data on the pharmacological effects of different drugs in poorly studied species, often with unique physiological characteristics, zoological medicine practitioners are often “flying blind”.

For example, a standing sedation for a laparoscopic procedure in an African elephant was produced with multiple injections of an α_2_ antagonist (detomidine) and opioid (butorphanol) [14]. Yet, little is known about how elephants metabolize these anesthetic drugs, whether there are age-related differences in their metabolism of such drugs, or how to determine which elephant is more likely to have a poor outcome to a particular anesthetic combination.

With the wide choice of anesthetics and sedatives available for both human and domestic species, the zoological medicine practitioner can create increasingly nuanced anesthesia protocols. While the goal is to administer each drug at an optimal dose with only the intended anesthetic or sedative effect, the paucity of information about these drugs in combination with each other, in a species where basic physiology is still poorly understood, means that the zoo veterinarian is often struggling to provide safe, evidence based anesthesia. Because balanced anesthesia protocols increase the number of drugs, the unstated risk is that there is increased probability of a drug-related adverse effect. For safety, attention should be given to selecting protocols with the maximum quality of anesthesia and the fewest number of administered agents. Publications of studies comparing anesthetic combination protocols where a premium is placed on reduction of drugs to give the optimal effects, should be a research priority in zoological medicine.

## 4. What Is the Issue (Why Does Polypharmacy Matter in Zoological Medicine?)

As noted, zoological medicine requires multiple educated, thoughtful, pharmacological decisions based on limited information for a given species. To paraphrase Dr. Murray Fowler, the father of modern zoological medicine: No presently available pharmaceutical agent is equally effective and safe for use with all 45,000+ vertebrate species for its approved indication [21]. Thus, zoological medicine veterinarians are, by necessity, very well versed in making reasonable inter-species pharmacological extrapolations [22]. General assumptions are made that non-domestic species drug metabolism can be approximated by closely related domestic species. For example, drug selection for a zebra might be expected to be similar to drugs used in horses with similar medical conditions.

However, while this assumption often does work, it also sometimes fails. The “issue” is deciding when the pharmacological differences are enough to be clinically relevant. For example, sedative combinations used in a horse rarely work in zebras, a species that has high sympathetic nervous system activity, whereas certain combinations of anesthetic agents that work in zebras would be deleterious, if not fatal, in domestic horses. The zoological medicine veterinarian must be knowledgeable not only about which drugs can be used together and how these drugs react with each other, but also about the basic physiology and even temperament of the non-domestic animal which may determine the effectiveness of particular drug combinations. Despite this, the full range of drug-drug interactions in the wide diversity of species in zoological medicine are truly difficult to predict. Polypharmacy, practiced in the zoological medicine context, thus has the potential to be a recipe for a pharmacological train wreck. To rephrase that, using the fewest number of drugs should be a goal.

## 5. What Are the Practical Solutions?

The first step towards a practical solution to polypharmacy in zoological medicine is recognizing the problem. This topic is seldom considered in zoological medicine. Every clinician is focused on treating and eliminating the clinical problems, yet is rarely looking for the negative effects of their pharmacological interventions. The clinician must learn to look for subtle signs of cumulative side effects and try to determine if the problems increase with the length of treatment. The zoological medicine practitioner must learn to recognize the risks associated with polypharmacy.

The next step is to adapt the process of “deprescribing”, i.e., decreasing the number of concurrently administered pharmaceuticals [23]. The goal is to actively remove unnecessary or potentially unwanted treatments administered to a given patient. Removing such drugs has been proven to decrease the incidence of adverse drug reactions, and improve the rate of medication compliance, thereby reducing the economic burden on both the patient and the health care provider [23].

Despite the availability of many tools in human medicine to minimize drug therapy related problems, little guidance currently exists for the process of deprescribing in general veterinary clinical practice. Various methods to reduce the risks of polypharmacy in human practice include patient education, physician education, and regulatory intervention. More research on deprescribing is a critical need in almost all branches of veterinary medicine because it will pave the way for better veterinary healthcare [23].

Specifically, zoological medicine practitioners should routinely review patients receiving multiple drug prescriptions. Such reviews can be conducted during annual preventative evaluations, when new drugs are prescribed, or when animals are evaluated while receiving long-term prescriptions. The list below, modified from Payne [1], provides items that zoological medicine veterinarians can consider prior to the administration of each drug or drug cocktails. Strong evidence of medication safety issues associated with polypharmacy are common in human medicine. Unfortunately, this is lacking in zoological medicine, in part due to the non-requirement for drug-drug interaction data during the drug approval process for animal health products, as well as a historical lack of awareness of polypharmacy [24].

### List of Indicators for Consideration Prior to Prescribing or Administering in a Zoological Medicine Patient

- Is there a valid indication/standard of practice for the drug in any species? (Does this standard also apply to this non-domestic species?)
- Is the medication expected to be effective for the condition? (Is there a measurable end-point for the therapy in the non-domestic species?)
- Is the dosage correct? (Are species-specific studies published? If not, what is the basis for the extrapolated dose?)
- Is the duration of therapy acceptable?
- Are the pharmaceutics of the drug practical?
- Are there clinically relevant, significant, drug-drug interactions?
- Is there unnecessary duplication with other drug(s)?

## 6. Summary

Polypharmacy is associated with drug–drug interactions that may lead to the development of new medical conditions, new adverse events, or to the worsening of existing diseases. We have highlighted, through several elephant examples, that the use of multiple pharmaceuticals in zoological medicine may be associated with minimal pharmacological or toxicological information for each prescribed drug. This lack of information is compounded when multiple drugs are used. Therefore, zoological medicine veterinarians need to carefully weigh the risks and benefits before prescribing and treating with multiple drugs. Recognizing the potential problem of polypharmacy in zoological medicine through this type of discussion is an important step. Having recognized the potential problem, actively looking for polypharmacy-associated negative side effects is essential, in addition to appropriately reporting them both to the pharmaceutical manufacturer and to the zoological medicine community.

The choice of drugs used in an individual zoological animal is ultimately an art based on science that can be modified to create safe and effective treatments resulting in positive outcomes. With the wide choice of drugs available for both human and domestic species, the zoological medicine practitioner can create increasingly nuanced treatment protocols. However, the unstated risk is that the probability of a drug related adverse event is in large part a function of the number of drugs used in the protocol. Attention should therefore be given to selecting the treatments with the fewest number of administered pharmacological agents. Veterinarians should also report all adverse events, including reduced or failed efficacy, to the drug sponsor to allow for pharmacovigilance trends to be noted and reported. Additionally, the publication of case studies describing drug protocols, where a premium is placed on the reduction of drugs to give the optimal effects or documentation of the negative effects of polypharmacy, should be a priority in zoological medicine.

Ultimately, it is the combination of limited pharmacokinetic, pharmacodynamic, and toxicology information in the non-domestic species that makes treating them inherently risky. However, the Hippocratic Oath is a guiding principal in all medical fields, including zoological medicine, “Above all, do no harm”. Multiple medications are sometimes required for optimal therapeutic treatment of some diseases or for administration of safely balanced anesthesia. Often the use of fewer drugs can accomplish the same outcome, while minimizing the problems of polypharmacy.
